# Comparison of face-down posturing with nonsupine posturing after macular hole surgery: a meta-analysis

**DOI:** 10.1186/s12886-019-1047-8

**Published:** 2019-01-28

**Authors:** Song Xia, Xin-yu Zhao, Er-qian Wang, You-xin Chen

**Affiliations:** 1 Department of Ophthalmology, Guizhou Provincial People’s Hospital, Guiyang, 550000 China; 2 Department of Ophthalmology, Peking Union Medical College Hospital, Chinese Academy of Medical Sciences, Beijing, 100730 China

**Keywords:** Macular hole, Face-down, Posturing, Meta-analysis

## Abstract

**Background:**

A few randomized controlled trials (RCTs) have evaluated face-down posturing (FDP) with the far less physically challenging nonsupine posturing (NSP) in the treatment of idiopathic full-thickness macular holes (MHs). The objective of our study was to evaluate the efficacy of postoperative posturing on the anatomical and functional outcomes of MH surgery.

**Methods:**

The PubMed, Embase, and Cochrane Central Register of Controlled Trials databases were searched from their earliest entries through December 2016 to identify the studies that had evaluated the effects of postoperative posturing with FDP or NSP for patients with MH surgery. The PRISMA guidelines were followed. The relevant data were analyzed using StataSE 12.0 software. The weighted mean difference (WMD), relative risk (RR) and their 95% confidence intervals (95% CIs) were used to assess the strength of the association.

**Results:**

Our search yielded 181 records from which 11 studies comprising 726 cases that had examined the effects of postoperative posturing with FDP for patients compared with NSP after MH surgery were included for review and analysis. Our meta-analyses showed that postoperative FDP could generally improve the overall MH closure rate compared to NSP (OR = 1.828, 95% CI: 1.063~3.143, *P* = 0.029). Subgroup analysis of the size of MH suggested a significant benefit of FDP for large MHs (≥400 μm) (OR = 4.361, 95% CI: 1.429~13.305, *P* = 0.010) while there was no difference in the MH closure rate for small MHs (< 400 μm) (OR = 1.731, 95% CI: 0.412~7.270, *P* = 0.453). Moreover, ILM peeling for large MHs could significantly increase the MH closure rate of the FDP group (OR = 2.489, 95% CI: 1.021~6.069, *P* = 0.045), while no difference existed for small MHs (OR = 3.572, 95% CI: 0.547~23.331, *P* = 0.184). Combined cataract surgery might not influence the MH closure rate under any circumstance (OR = 0.513, 95% CI: 0.089~2.944, *P* = 0.454).

**Conclusion:**

Based on all the available evidence, our study found that FDP after MH surgery could generally improve the overall MH closure rate compared to NSP. For MHs larger than 400 μm, ILM peeling combined with FDP could significantly increase the MH closure rate. Combined cataract surgery might not influence the MH closure rate.

## Introduction

Idiopathic full-thickness macular holes (MHs) are one of the most significant causes of visual loss. MHs have an estimated incidence of 7.8 per 1000,000 individuals, and they commonly affect elderly females [1–3]. In 1991, Kelly and Wendel [4] first described the traditional surgical intervention for idiopathic MHs, which comprised pars plana vitrectomy, removal of the adherent cortical vitreous, peeling of epiretinal membranes and intraocular tamponade with gas, followed by strict face-down posturing (FDP) for one week. With the significant progress in surgical techniques, such as internal limiting membrane (ILM) peeling and combined cataract surgery, hole closure rates have improved from 58 to 100%, as reported in recent studies [4–6].

However, some controversies regarding the postoperative position still exist. A few randomized controlled trials (RCTs) have evaluated FDP with the far less physically challenging nonsupine posturing (NSP) in the treatment of idiopathic full-thickness MHs, and some of them suggested a significant benefit of FDP, while others suggested that FDP is unnecessary [6–10]. As there were heterogeneous study methods and varying surgical techniques, current studies could not provide sufficient data to achieve firm conclusions about the optimal postoperative position, and some authors held the opinion that equivalent closure rates could be achieved with NSP [8, 11, 12]. Despite NSP receiving favorable reviews, the lack of clear superiority between FDP and NSP has prevented NSP from entering into clinical practice, as demonstrated by a recent survey of American Society of Retina Specialists, which revealed that 95% of retinal surgeons still incorporate FDP in the treatment of MHs [13, 14]. If high hole closure rates can be achieved without FDP, MH surgery can be made available to many patients who were previously unable or unwilling to tolerate the postoperative FDP. Additionally, some severe complications such as ulnar nerve palsies, thrombophlebitis and pulmonary embolism could also be avoided [15, 16].

A previous meta-analysis [17] was performed to evaluate the efficacy of postoperative posturing on the anatomical and functional outcomes of MH surgery. They made a subgroup analysis of the size of the MH and drew the following conclusions: FDP significantly improved the success rate of the surgery for MH larger than 400 μm, while it was unnecessary for MH less than 400 μm. Although this was a scientific and rigorous study, some limitations still existed: (1) Only 4 previous RCTs were included and other available and recent data were not pooled; and (2) Other adjunct treatments, such as inner limiting membrane (ILM) peeling and combined cataract surgery, were not considered. Hence, we performed this meta-analysis, which included all the available data and took all the influential factors into consideration to evaluate the superiority of FDP over NSP following MH surgery in order to provide a reference for the decision-making of ophthalmologists.

## Methods

This systematic review and meta-analysis was performed strictly according to the guidelines given by the ‘Preferred Reporting Items for Systematic Reviews and Meta-Analysis (the ‘PRISMA’ statement)’ [18].

### Search strategy

Two independent researchers (SX and XYZ) searched the PubMed, Embase, and Cochrane Central Register of Controlled Trials databases. Data were last updated in June 2017. The following keywords or corresponding Medical Subject Headings (Mesh) were used: “macular hole”, “position”, “posturing”, “face-down” and “supine”. The references of the included studies were also screened to further identify related articles. No language limitation was imposed.

#### Inclusion criteria and exclusion criteria

Inclusion criteria were as follows: (1) Participants were people with macular holes that required surgical intervention; (2) The intervention was macular hole surgery; (3) FDP was compared with NSP; (4) The MH closure rate, best-corrected visual acuity or more were the outcomes; and (5) The methodology needed to be a prospective study, a case-controlled study or a cohort study.

Exclusion criteria were as follows: (1) Other differences existed between the case group and control group in addition to the postoperative face position; (2) There was insufficient data to estimate an odds risk (OR) or weighted mean difference (WMD); (3) The study was an animal study or subjects were cadavers; and (4) There were redundant publications.

### Data extraction and assessment of methodological quality

After filtering titles and abstracts, then reviewing the full texts of potentially related articles, the studies which fulfilled eligibility criteria and failed the exclusion criteria were included. SX and XYZ extracted and collated the relevant data, including the first author’s name, publication year, design, sample size, group size, average age, duration of FDP, details of the surgical procedure, intra- and postoperative evaluating parameters, and follow-up periods. The corresponding authors of included studies would be contacted if requisite data were unavailable. The methodological quality of each included study was evaluated by 12-item scale [19]; a score of 7 or more was high quality, 4 to 7 was moderate quality, less than 4 was low quality. Kappa text was used to evaluate the disagreements and consensus was achieved by discussion with the corresponding author (YXC).

#### Statistical methods

StataSE 12.0 software (StataCorp, College Station, TX, USA) was used to perform the meta-analysis. In this meta-analysis, continuous data was described by the weighted mean difference (WMD) and its 95% confidence interval (CI), dichotomous data used odds risk (OR) and its 95% CI. The statistical heterogeneity was assessed with a Chi-squared test and *I*^*2*^. If heterogeneity was low (*P*>0.1, *I*^*2*^<50%), a fixed-effect model was used; for substantial heterogeneity (*P*<0.1, *I*^*2*^>50%), sensitivity analysis and subgroup analyses were conducted to identify the source of the heterogeneity. If the heterogeneity could not be eliminated, a random-effects model was used when the result of the meta-analysis had clinical homogeneity or a descriptive analysis was used. A stratified subgroup analysis, which evaluated the possible influencing factors, was also conducted.

Publication bias was evaluated with a Begg’s funnel plot. A value of *P* < 0.05 was considered to indicate statistical significance [20] .

#### Source of funding

No external funding was received in support of this study.

## Results

### Study characteristics

**Fig. 1:**
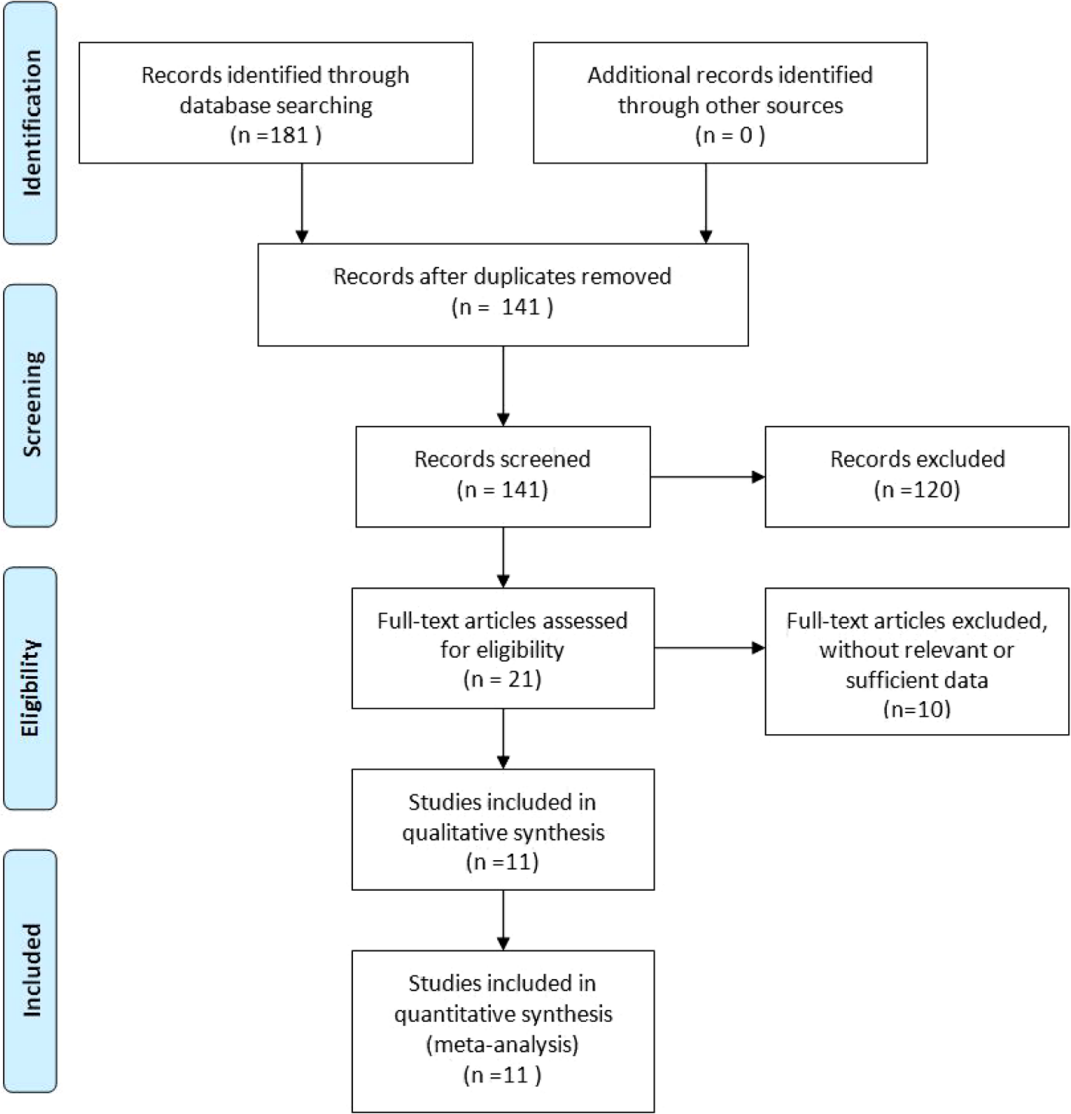
Flowchart depicting the selection of included studies

One hundred eighty-one articles were initially identified for this meta-analysis. After removing 40 duplicate studies, screening titles and abstracts, and removing 120 unrelated articles, 11 studies [7–12, 14, 21–24] were finally included in this meta-analysis (Fig. 1). The cumulative sample size of 742 MH surgeries comprised 396 eyes in the FDP group and 346 eyes in the no FDP group. In each study, the demographic characteristics of the two groups were similar. The main characteristics of the included studies are summarized in Table 1 and the literature-exclusion procedures are depicted in Fig. 1. The results of 12-item scale showed that the average quality score of the included studies was 8.18 and all of them were of high quality (Table 2). The inter-rater agreement was excellent between the investigators (κ = 0.75).

**Table 1:** Main characteristics of the included studies

First author	Publication year	Design	MH stage	Sample size(Patients)	Group size(Eyes)	Average age	Gender ratio (Male/female)		Duration of FDP	Details of the surgical procedure	Intra- and postoperative evaluating parameters	Follow-up periods
					FDP/NSP	FDP/NSP	FDP	NSP				
Alberti, M	2016	RCT	Stage 2 3 4	68	34/34	(69.8 ± 6.9) / (69.3 ± 5.7)	3/12	9/6	3 days	23-G PPV with the fill of 15%C3F8 and the peel of ILM.	Postoperative BCVA, MH closure rate, complications	3 months
Alberti, M	2015	Retrospective study	Stage 2 3 4	117	66/56	(70.4 ± 6.8) / (69.5 ± 5.8)	22/44	21/36	3 days	23-G PPV with the fill of 15%C3F8 and the peel of ILM.	Postoperative BCVA, MH closure rate, complications	6 months
Feist, RM	2014	Retrospective study	NA	82	57/25	NA	27/35	11/14	3 days	PPV with the fill of C3F8 or SF6 and the peel of ILM.	Postoperative BCVA, MH closure rate, complications	NA
Forsaa, VA	2013	Retrospective study	Stage 2 3 4	64	33/34	(68.4 ± 9.0) / (71.7 ± 6.8)	8/2	4/5	3 days	23-G PPV with the fill of 22–30%SF6 or 16–20%C2F6 and the peel of ILM.	Postoperative BCVA, MH closure rate, complications	4.7–19.8 months
Yorston, D	2012	RCT	Stage 2 3 4	30	16/14	(71.1 ± 5.9) / (68.0 ± 5.6)	1/15	0/14	10 days	20-G PPV with the fill of 14%C3F8 and the peel of ILM.The phacoemulsification with introcular lens insertion was carried out.	Postoperative BCVA, MH closure rate, complications	6 months
Lange, CA	2012	RCT	Stage 2 3 4	30	15/15	(66.8 ± 5.9) / (71.0 ± 6.2)	7/11	11/6	10 days	20-G PPV with the fill of 14%C3F8 and the peel of ILM.	Postoperative BCVA, MH closure rate, complications	6–8 weeks
Tadayoni, R	2011	RCT	≤400 μm	69	34/35	(67.31 ± 6.46) / (66.31 ± 6.51)	11/23	15/20	10 days	PPV with the fill of 17%C2F6.	Postoperative BCVA, MH closure rate, complications	3 months
Guillaubey, A	2008	RCT	Stage 2 3 4	144	72/78	(55.7 ± 9.9) / (55.7 ± 7.4)	25/47	27/51	10 days	20-G PPV with the fill of 14%C3F8, 17%C2F6 or 20%SF6 and the peel of ILM.When needed, 62/150 had phacovitrectomy.	Postoperative BCVA, MH closure rate, complications	6 months
Tranos, PG	2007	RCT	Stage 2 3 4	41	25/16	NA	NA	NA	10 days	20-G PPV with the fill of 16%C3F8 and the peel of ILM.	Postoperative BCVA, MH closure rate, 25-Visual Function Questionnaire(VFQ-25) and complications	4 months
Simcock, PR	2001	Non-randomised historically controlled study	Stage 2 3	33	13/20	NA	2/11	5/15	10 days	PPV with the fill of 20% C2F6.phacoemulsification with introcular lens insertion was combined.	Postoperative BCVA, MH closure rate, complications	3 months
Szurman, P	2000	Consecutive case control study	Stage 2 3 4	50	25/25	(67 ± 5.6) / (67 ± 4.2)	6/19	6/19	14 days	PPV with fill of 20%SF6 or 16% C3F8.	Postoperative BCVA, MH closure rate, complications	6–12 months

*RCT* Randomized controlled trial. *MH* Macular hole. *FDP* Face-down posturing. *NSP* Non-supine posturing. *BCVA* Best corrected vision acuity. *NA* Not available

**Table 2:** 12-item scale critical appraisal scores

Author	12-item scale critical appraisal score												
	1	2	3	4	5	6	7	8	9	10	11	12	Quatily
Guillaubey, A 2008	N	Y	N	N	N	Y	Y	Y	Y	Y	Y	Y	High
Tranos, PG 2007	N	Y	N	N	N	Y	Y	Y	Y	Y	Y	Y	High
Lange, CA 2012	Y	Y	N	N	N	Y	Y	Y	Y	Y	Y	Y	High
Yorston, D 2012	N	Y	N	N	N	Y	Y	Y	Y	Y	Y	Y	High
Simcock, PR 2001	N	Y	N	N	N	Y	Y	Y	Y	Y	Y	Y	High
Tadayoni, R 2011	N	Y	N	N	N	Y	Y	Y	Y	Y	Y	Y	High
Szurman, P 2000	N	Y	N	N	N	Y	Y	Y	Y	Y	Y	Y	High
Alberti, M 2015	N	Y	N	N	N	Y	Y	Y	Y	Y	Y	Y	High
Feist, RM 2014	N	Y	N	N	N	Y	Y	Y	Y	Y	Y	Y	High
Forsaa, VA 2013	N	Y	N	N	N	Y	Y	Y	Y	Y	Y	Y	High
Alberti, M 2016	Y	Y	N	Y	N	Y	Y	Y	Y	Y	Y	Y	High

12-item scale criteria: (1)Method of randomization; (2)Concealed allocation; (3)Patient blinding; (4)Provider blinding; (5)Outcome assessor blinding; (6)Drop-out rate; (7)Patient allocated as plan; (8)Free of selective outcome reporting; (9)Same baseline; (10)Co-interventions avoided or similar; (11)Acceptable compliance; (12)Same time of outcome assessment. Y=Yes, N=No, A trial with a score of 7 or more was considered high quality, more than four but no more than seven was considered moderate quality, and no more than four was considered low quality

### Macular hole closure rate

Together, eleven studies [7–12, 14, 21–24] comprised 726 cases, including 396 eyes in the FDP group and 346 eyes in the NSP group, which described the MH closure rate, respectively.

**Fig. 2:**
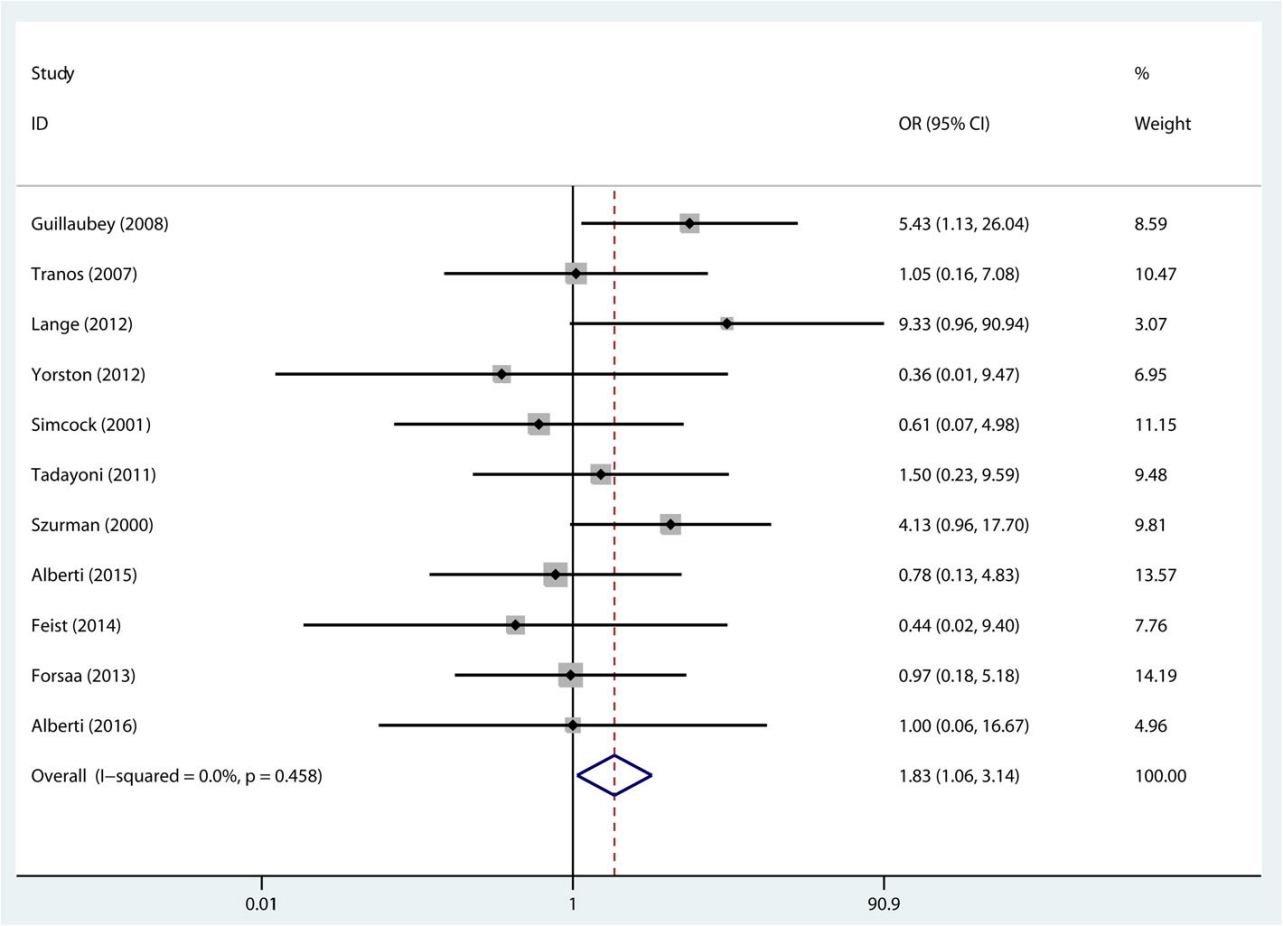
Comparison of the macular hole closure rate in the face-down posturing group and nonsupine posturing group

For the overall MH closure rate, a fixed-effects model was used as no heterogeneity was detected (*P* = 0.458, *I*^*2*^ = 0%). The pooled results showed that the MH closure rate in the FDP group was significantly higher than that of the NSP group (OR = 1.828, 95% CI: 1.063~3.143, *P* = 0.029, Fig. 2).

**Fig. 3:**
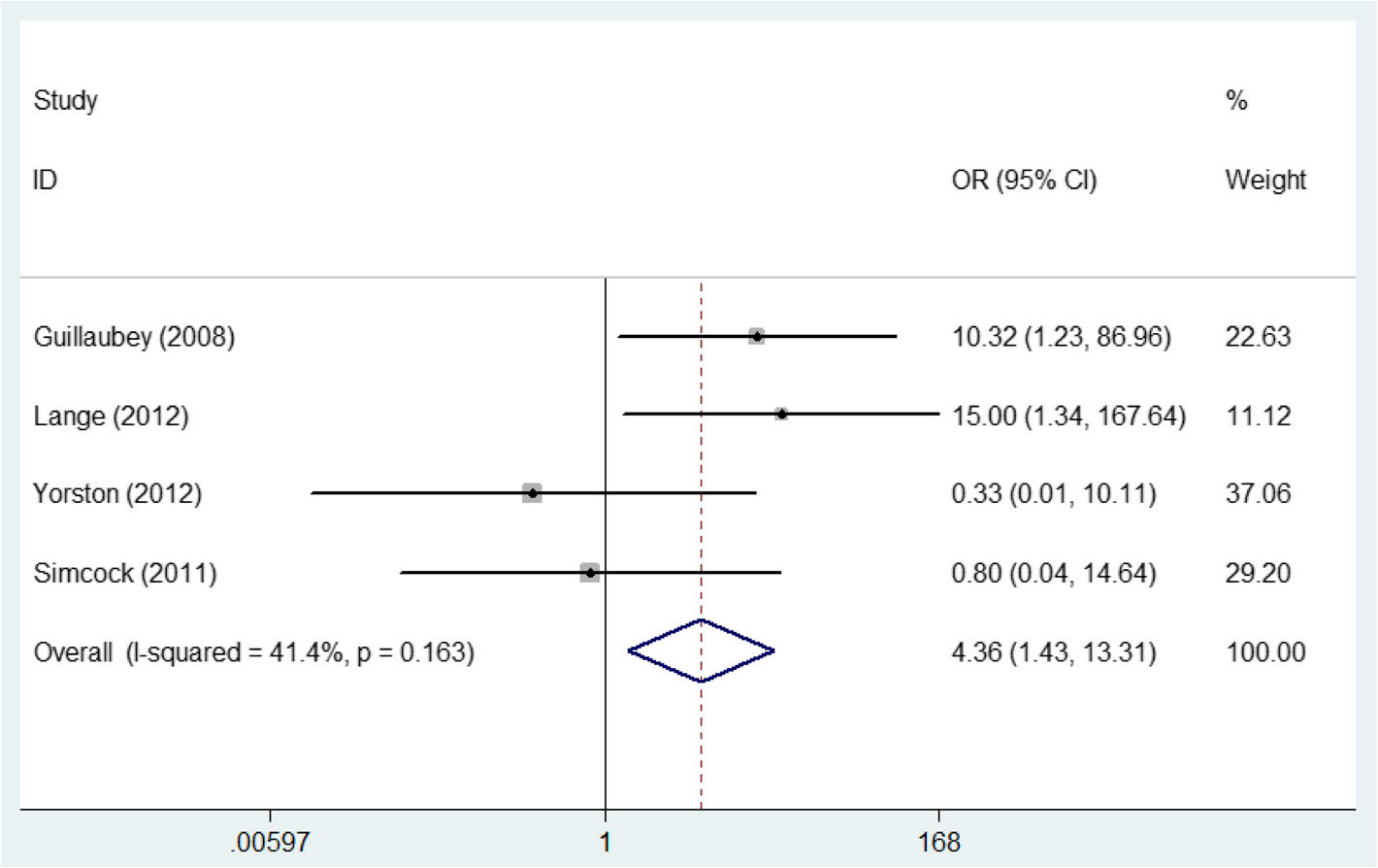
Comparison of the macular hole closure rate with a size greater than 400 μm in the face-down posturing group and nonsupine posturing group

**Fig. 4:**
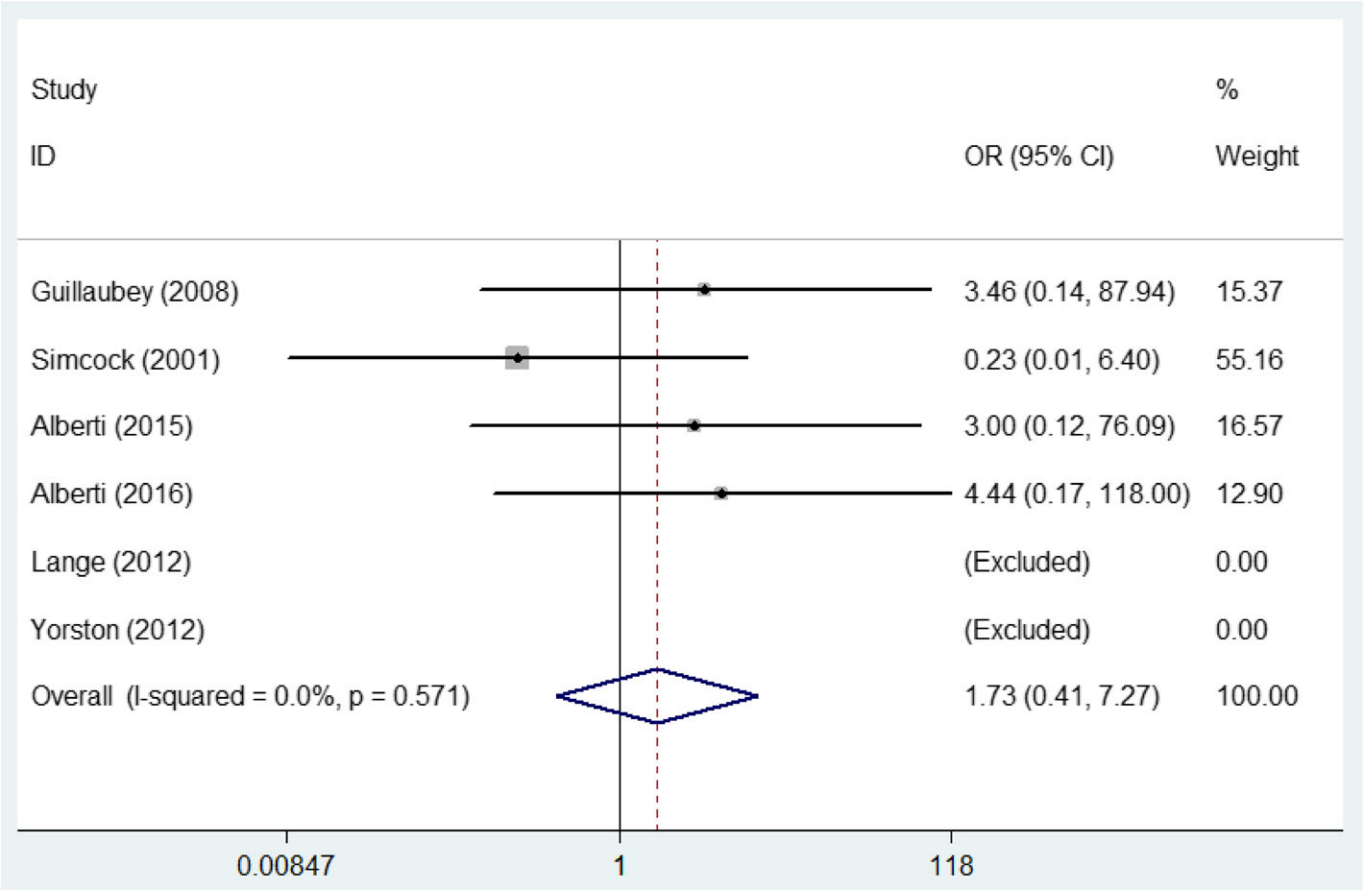
Comparison of the macular hole closure rate with the macular hole size smaller than 400 μm in the face-down posturing group and nonsupine posturing group

As 400 μm (narrowest width at largest aperture of MH) was defined as the cut-off point when classifying MHs [25], we used it to divide the included patients into two subgroups for further analysis. For MHs with a size more than 400 μm, the pooled result according to the fixed-effects model (*P* = 0.163, *I*^2^ = 41.4%) manifested that the MH closure rate in the FDP group was significantly higher than that of the NSP group (OR = 4.361, 95% CI: 1.429~13.305, *P* = 0.010, Fig. 3). For MHs smaller than 400 μm, the meta-analysis pooled results according to the fixed-effects model (*P* = 0.571, *I*^2^ = 0%) indicated no significant difference in the MH closure rate between the two groups (OR = 1731, 95% CI: 0.412~7.270, *P* = 0.453, Fig. 4).

As several previous studies have reported that MH surgery might benefit from ILM peeling [26–28], which strengthens the treatment, a comprehensive evaluation of this factor was conducted. Regardless of the size of the MH, the subgroup analysis with the fixed-effects model (*P*_*overall*_ = 0.518, *I*_*overall*_^2^ = 0%) showed that ILM peeling might not influence the MH closure rate between the FDP group and the NSP group (*P*_*peeling*_ = 0.096, *P*_*none*_ = 0.978, *P*_*overall*_ = 0.130, Table 3). For MHs larger than 400 μm, the forest plots created with the fixed-effects model (*P*_*overall*_ = 0.094, *I*_*overall*_^2^ = 46.9%) indicated that peeling the ILM significantly increased the MH closure rate of the FDP group compared to the NSP group, while no difference between the two groups was found when the surgery was performed without ILM peeling (*P*_*peeling*_ = 0.045, *P*_*none*_ = 0.880, *P*_*overall*_ = 0.060, Table 3). However, for MHs smaller than 400 μm, the meta-analysis pooled according to the fixed-effects model (*P* = 0.571, *I*^2^ = 0%) indicated that whether there was peeling of the ILM or not, there was no significant difference in the MH closure rate between the two groups (*P*_*peeling*_ = 0.184, *P*_*none*_ = 0.390, *P*_*overall*_ = 0.453, Table 3).

**Table 3:** Comparison of macular hole closure rate between two groups with or without internal limiting membrane peeling

Outcome	Type of subgroup	No. of studies	Sample size		With ILM Peeling				Without ILM Peeling				*Selected model*
			Face-down group	No face-down group	OR	95% CI		*P* value	OR	95% CI		*P* value	
MH closure rate	Overall	10	371	321	1.744	0.906	3.356	0.096	1.020	0.258	4.026	0.978	Fixed-effect model
	MH ≥ 400 μm	6	107	101	2.489	1.021	6.069	0.045	0.080	0.044	14.643	0.880	Fixed-effect model
	MH<400 μm	6	115	110	3.572	0.547	23.331	0.184	0.235	0.009	6.401	0.390	Fixed-effect model

*MH* Macular hole, *ILM* Internal limiting membrane, *CI* Confidence interval, *OR* Odds risk

Additionally, as some authors previously demonstrated the benefit of phacovitrectomy with intraocular lens implants in MH surgery [8, 29], we conducted another subgroup analysis focused on combining MH surgery with or without phacovitrectomy and intraocular lens implants. The overall subgroup analysis (fixed-effects model, *P*_*overall*_ = 0.565, *I*_*overall*_^2^ = 0%) showed that phacovitrectomy and an intraocular lens implant might not influence the MH closure rate between the FDP group and the NSP group (*P*_*combine*_ = 0.453, *P*_*none*_ = 0.471, *P*_*overall*_ = 0.737, Table 4). For MHs larger than 400 μm, the meta-analysis conducted with a fixed-effects model (*P*_*overall*_ = 0.176, *I*_*overall*_^2^ = 36.8%) indicated that phacovitrectomy and intraocular lens implants did not influence the MH closure rate between the two groups (*P*_*combine*_ = 0.573, *P*_*none*_ = 0.370, *P*_*overall*_ = 0.615, Table 4). For MHs smaller than 400 μm, the pooling results with a fixed-effects model (*P*_*overall*_ = 0.405, *I*_*overall*_^2^ = 0%) also showed that phacovitrectomy and intraocular lens implants did not influence the MH closure rate between the two groups (*P*_*combine*_ = 0.390, *P*_*none*_ = 0.273, *P*_*overall*_ = 0.674, Table 4).

**Table 4:** Comparison of macular hole closure rate between two groups with or without phacovitrectomy with intraocular lens implant

Outcome	Type of subgroup	No. of studies	Sample size		With phacovitrectomy with intraocular lens implant				Without phacovitrectomy with intraocular lens implant				*Selected model*
			Face-down group	No face-down group	OR	95% CI		*P* value	OR	95% CI		*P* value	
MH closure rate	Overall	8	259	214	0.513	0.089	2.944	0.454	1.337	0.608	2.942	0.471	Fixed-effect model
	MH ≥ 400 μm	5	66	62	0.539	0.063	4.629	0.573	1.702	0.533	5.438	0.370	Fixed-effect model
	MH<400 μm	5	78	77	1.417	0.279	7.192	0.390	3.360	0.363	36.346	0.273	Fixed-effect model

*MH* Macular hole, *CI* Confidence interval, *OR* Odds risk

### Ideal visual acuity improvement

In total, six studies [7–9, 12, 14, 23], including 144 eyes in the FDP group and 152 eyes in the NSP group, described the rate of ideal postoperative visual acuity improvement.

**Fig. 5:**
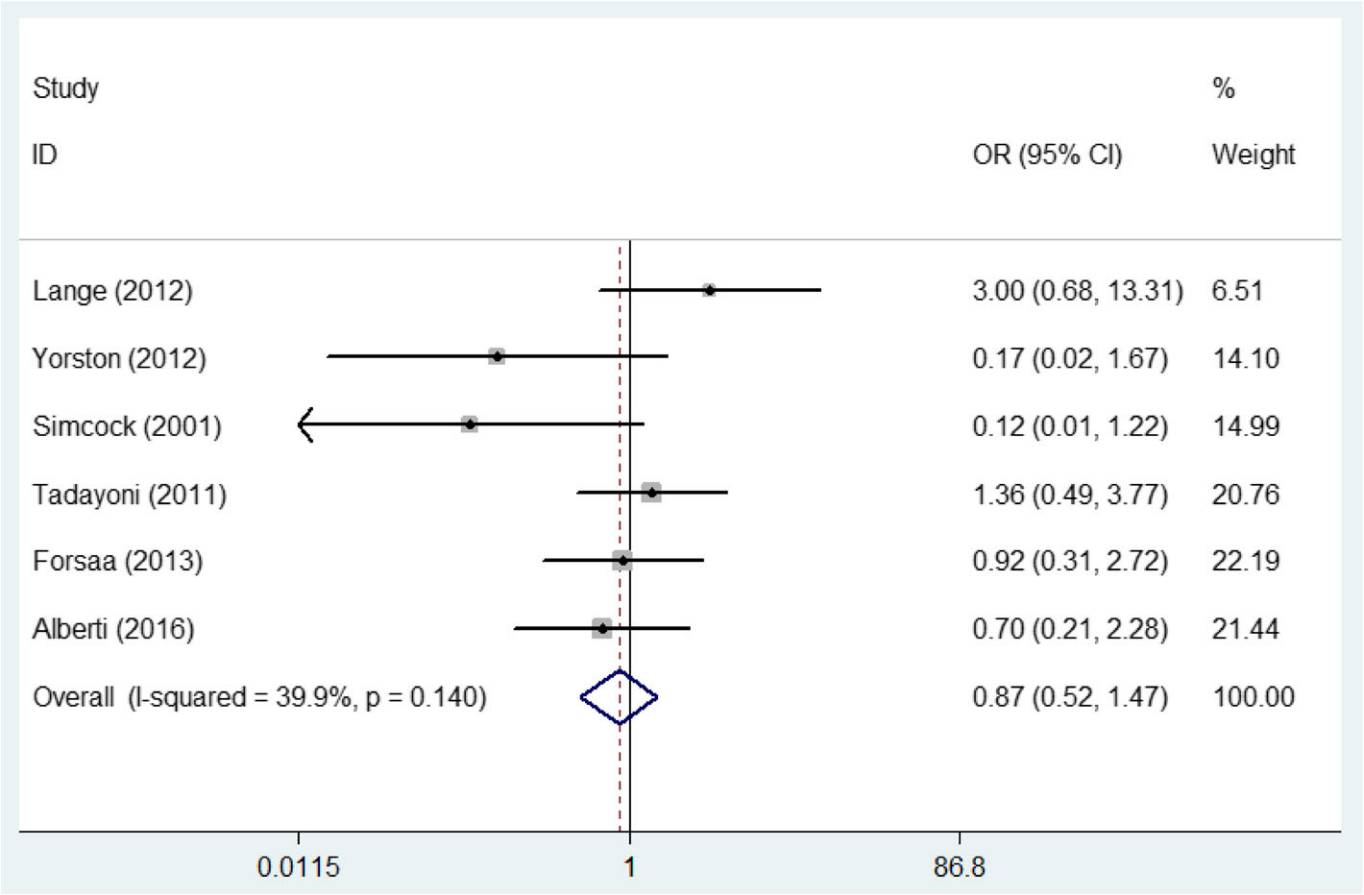
Comparison of the ideal visual acuity improvement rate in the face-down posturing group and nonsupine posturing group

For the overall ideal VA improvement rate, the meta-analysis pooled with a fixed-effect model (*P* = 0.140, *I*^2^ = 39.9%) indicated no significant difference in the ideal VA improvement rate between the two groups (OR = 0.873, 95% CI: 0.521~1.466, *P* = 0.609, Fig. 5).

For MHs with a size more than 400 μm, the meta-analysis using the random-effects model (*P* = 0.039, *I*^2^ = 76.5%) manifested no significant difference in the ideal VA improvement rate between the two groups (OR = 0.727, 95% CI: 0.017~31.794, *P* = 0.868). For MHs smaller than 400 μm, the forest plots resulting from the fixed-effects model (*P* = 0.236, *I*^2^ = 28.9%) also indicated no significant difference in the ideal VA improvement rate between the two groups (OR = 0.466, 95% CI: 0.076~2.847, *P* = 0.408).

### Publication bias

Begg’s test showed that publication bias did not affect our analysis (*P* = 0.175, continuity corrected).

## Discussion

Our study showed that postoperative FDP could generally improve the overall MH closure rate compared to the NSP group. Subgroup analysis of the size of the MH suggested a significant benefit of FDP for large MHs (≥400 μm), while no difference in the MH closure rate was observed for small MHs (< 400 μm). Moreover, ILM peeling for large MHs could significantly increase the MH closure rate of the FDP group, while no difference existed for small MHs. Combined cataract surgery might not influence the MH closure rate under any circumstances. Additionally, the ideal visual improvement rate was not influenced by postoperative positioning.

Postoperative positioning after MH surgery is mostly influenced by intraocular gas tamponade, which has two major properties: surface tension and buoyancy. The surface tension depends on the viscosity of the tamponade product. Buoyancy depends on density, and it is maximal at the apex of the gas bubble. Buoyancy can remove the subretinal fluid to reattach the retina [10, 30, 31], which is why the postoperative position after retinal detachment is crucial. The actual mechanism for how the intraocular gas tamponade facilitates MH closure is still debated [6, 24]. The generally accepted opinion was that the gas bubble could isolate the macula from the vitreous fluid and keep it dry, thereby providing a scaffold to support the formation of a bridging preretinal membrane, rather than exerting upward buoyancy forces on the macular hole [31]. If this is correct, a large gas bubble should be sufficient to keep the macula dry in all but the supine position [6, 32–35]. However, the results of our meta-analysis suggested that postoperative FDP could generally improve the overall MH closure rate compared to NSP, which might hint that the upward mechanical force of buoyancy is still beneficial, especially for large MHs (≥400 μm). These results are regarded as highly reliable as the heterogeneity was very low, and the comparisons were all carried out with fixed-effects models.

The effect of ILM peeling on MH surgery is still under debate [27]. Previous studies suggested that it could lead to better healing of MHs and prevent later reopening [36, 37]. After pooling all the available data from previous studies, we found a positive relationship between the size of idiopathic MHs and the benefit of ILM peeling. The subgroup analysis of our study suggested that ILM peeling was advantageous for MHs larger than 400 μm and was unnecessary for smaller idiopathic MHs.

The nonclosure of an MH is often attributed solely to poor positioning compliance; however, a recent study reported that equal attention should be shifted toward achieving maximal gas tamponade. Indeed, additional gas injection was regarded as an effective intervention to repair an unclosed MH after an unsuccessful primary vitrectomy [38]. Some authors have suggested that combined cataract surgery could not only facilitate a more complete vitrectomy, enabling injection of a larger gas volume when compared with the phakic eye, but also eliminate the need for a technically challenging cataract surgery in the vitrectomized eye, which is due to a lack of vitreous support, and may result in an unstable anterior chamber depth and various pupil sizes [39, 40]. However, the forest plots in our study indicated that combined cataract surgery might not influence the MH closure rate under any circumstance.

Postoperative VA is influenced by many factors such as preoperative VA, duration of follow-up and combination with cataract surgery or not. In total, six studies provided the rate of ideal postoperative VA improvement, which enabled us to analyze the pooled OR. The forest plots of our meta-analysis indicated that the ideal visual improvement rate was not influenced by postoperative positioning or the size of the MH.

To the best of our knowledge, this is the first meta-analysis which included all the available data and considered all the influential factors, such as the size of the MH, ILM peeling and combined cataract surgery, to re-evaluate superiority between FDP and NSP following MH surgery. The heterogeneity of our analysis was satisfactory and the results are reliable. Our study, therefore, might provide valuable information for ophthalmologists. However, it has the following limitations: (1) The inconsistent duration of FDP, which varied from 3 days to 14 days, might have potentially influenced the results of our study; (2) Although we pooled the data from all the available studies to achieve the results in a reliable way, the final sample size was still relatively small, which means more research of high quality should be carried out; and (3) There were insufficient data to analyze the effect of various types of intraocular gas and further research should focus on this point as it might also affect the choice of postoperative positioning.

## Conclusions

Based on all the available evidence, FDP after MH surgery could generally improve the overall MH closure rate compared to NSP. For MHs larger than 400 μm, ILM peeling combined with FDP could significantly increase the MH closure rate. Combined cataract surgery might not influence the MH closure rate under any circumstances. Further well-conducted RCTs are needed to verify our findings.

## Abbreviations

BCVA: Best-corrected visual acuity
CI: confidence interval
FDP: Face-down posturing
ILM: Inner limiting membrane
MH: Macular hole
NSP: Nonsupine posturing
OR: Odds risk
RCT: Randomized controlled trial
VA: Visual acuity
WMD: Weighted mean difference
